# Luminophore and Magnetic Multicore Nanoassemblies for Dual-Mode MRI and Fluorescence Imaging

**DOI:** 10.3390/nano10010028

**Published:** 2019-12-20

**Authors:** Lénaïc Lartigue, Marina Coupeau, Mélanie Lesault

**Affiliations:** Université de Nantes, CNRS, CEISAM UMR 6230, F-44000 Nantes, France; Marina.Coupeau@univ-nantes.fr (M.C.); melanie.lesault@etu.univ-nantes.fr (M.L.)

**Keywords:** magnetic multicore nanoparticles, magnetic interaction, MRI contrast agent, fluorescence imaging, dual-mode imaging

## Abstract

Nanoassemblies encompass a large variety of systems (organic, crystalline, amorphous and porous). The nanometric size enables these systems to interact with biological entities and cellular organelles of similar dimensions (proteins, cells, …). Over the past 20 years, the exploitation of their singular properties as contrast agents has led to the improvement of medical imaging. The use of nanoprobes also allows the combination of several active units within the same nanostructure, paving the way to multi-imaging. Thus, the nano-object provides various additional information which helps simplify the number of clinical procedures required. In this review, we are interested in the combination between fluorescent units and magnetic nanoparticles to perform dual-mode magnetic resonance imaging (MRI) and fluorescent imaging. The effect of magnetic interaction in multicore iron oxide nanoparticles on the MRI contrast agent properties is highlighted.

## 1. Introduction

**Figure 1:**
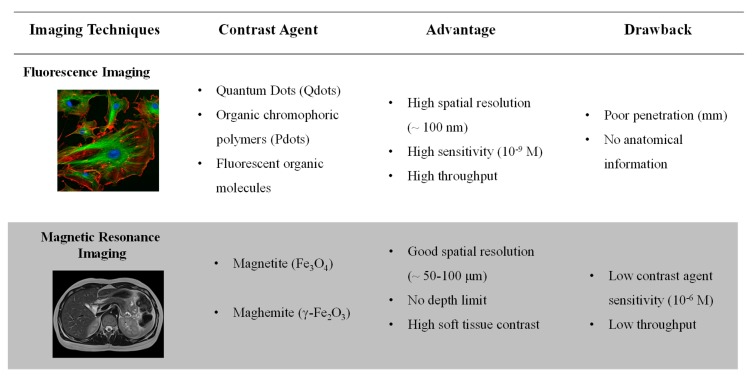
Fluorescence imaging vs. magnetic resonance imaging (MRI). Comparison between both imaging techniques showing their complementarity.

Medical imaging allows for non-invasive anatomical or functional diagnostics to detect dysfunctions or specific diseases. Physical principles of the considered technique determine the spatial resolution, the sensitivity level and the type of tissues or biological phenomena to be detected [1]. Among the most commonly used in clinics, we can mention X-ray computed tomography (CT scan), magnetic resonance imaging (MRI), ultrasound imaging, and nuclear imaging (single photon emission computed tomography, SPECT or positron emission tomography, PET) [1,2]. For all imaging techniques, contrast agents (i.e., active units) are generally essential and the use of nano-objects suggests promising perspectives [3]. Indeed, nano-objects enable the combination of different active units inside the same system. Thus, the development of multimodal nanoprobes made of various imaging units can advantageously multiplex images to avoid false-positive results, thereby providing earlier and more reliable diagnosis. Among the different possible combinations, growing interest has been shown in nanosystems comprising magnetic nanoparticles (MNPs) for MRI and luminescent entities for fluorescent imaging in recent years (Figure 1) [4]. Luminescence imaging, thanks to its multiple-label possibility, its high sensitivity and spatial resolution, is widely used to follow biological processes or in histopathology. However, extinction phenomena (diffusion and absorption of light by tissue) limit the depth penetration of this imaging technique. By contrast, MR present an unlimited depth penetration and greater soft-tissue contrast. Nevertherless, the low sensitivity of the MR technique makes it difficult to distinguish benign from malignant disease even with long acquisition time. So, combining these two safety techniques (using non-ionizing radiation) allows advantage to be taken of the high sensitivity and spatial resolution of luminescence imaging, associated to the good spatial resolution and deep tissue penetration of MRI. This combination is particularly interesting for correlating in vitro monitoring and in vivo tracking [5]. 

This review proposes to compare the different approaches envisaged to design dual-mode nanomaterials. A special emphasis is made on nanosystems embedding multicore magnetic nanoparticles. First, descriptions of the techniques and the different functional units are presented. Then, effects of self-assemblies magnetic iron oxide nanoparticles are discussed. Finally, the strengths and weaknesses are assessed of the three methods leading to magneto-fluorescent nanosystems: (1) association by covalent bonding; (2) encapsulation in matrices; (3) dispersion in nanoassemblies.

## 2. Active Units

### 2.1. Magnetic Entities for Magnetic Resonance Imaging (MRI)

**Figure 2:**
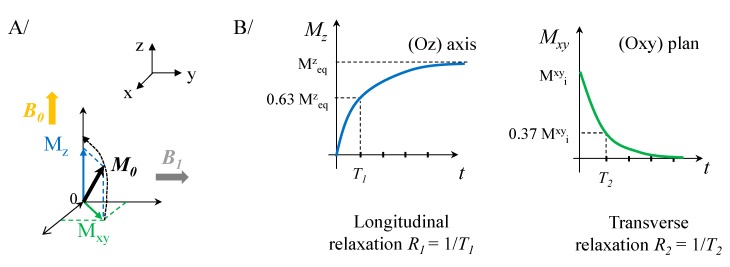
Schematic representation of the MRI. (**A**) Relaxation of protons under the action of an external magnetic field, *B_0_*, and radiofrequency pulse *B_1_*. (**B**) Return to the equilibrium position of total magnetization, *M_0_*, along the (Oz) axis and the (Oxy) plane.

MRI is a non-invasive technique using radio frequency (RF) pulses and strong magnetic fields to create images of internal organs and structures. MRI is based on the nuclear magnetic resonance of the hydrogen nuclei (proton) mainly those of water, which is abundant in most organs. The relaxation mechanism of these protons is explained in Figure 2A.

**Figure 3:**
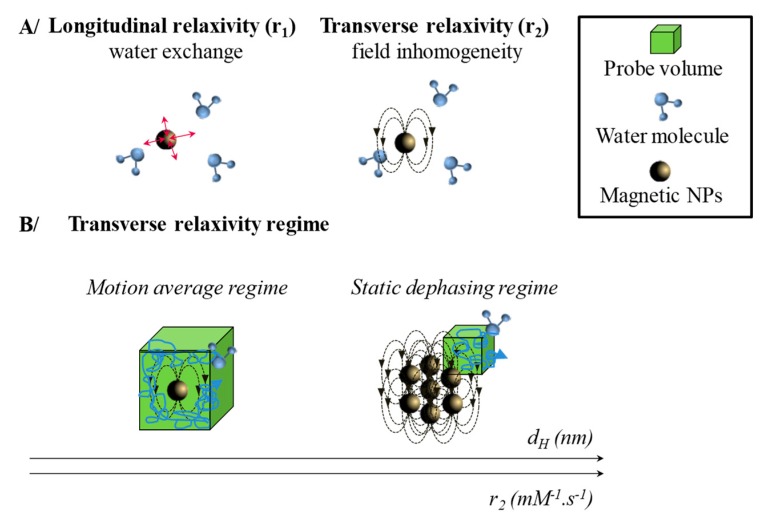
Interaction of magnetic system with water protons during an MRI experiment. (**A**) Schematization of the effects of magnetic nanoparticles (MNPs) on longitudinal and transverse relaxivity. (**B**) Probed volume by a water molecule during an MRI experiment depending on the size of the assembly. Consequence on the transverse relaxivity value and the associated model.

Hydrogen atoms have only one nuclear spin that induces a nuclear magnetic moment. In the presence of an external static magnetic field *B_0_*, the spin magnetic moments of protons, represented by a total magnetic moment, denoted *M_0_*, will precess around the direction of the field *B_0_*. A very short perpendicular RF *B_1_* pulse tilts the magnetic moment of protons in (Oz) plane. Their relaxation to the initial state is measured after the pulse. Return to the equilibrium position (i.e., *M_0_* in the same direction as *B_0_*) can be decomposed into two components *M_z_* and *M_xy_* (Figure 2B). *M_z_*, the component along the axis (Oz), which is associated with the longitudinal relaxation *R_1_* and the relaxation time *T_1_* (*R_1_* = 1/*T_1_*). The *M_xy_* component in the plane (Oxy) is related to the transverse relaxation *R_2_* and the relaxation time *T_2_* (*R_2_* = 1/*T_2_*). The longitudinal relaxation *R_1_* corresponds to a spin-lattice relaxation, i.e., exchange energy of the spins with the external medium [6]. The transverse relaxation *R_2_* corresponds to a spin-spin relaxation due to interactions between spin-magnetic dipole moments (Figure 3A). This transverse relaxivity is also sensitive to local magnetic field heterogeneities induced by species (tissues or materials placed within the field) possessing different magnetic susceptibility [6]. This local inhomogeneities produce, in addition to spin-spin relaxation, an additional dephasing fields. Both together induce an effective transverse relaxation called *T*_2_* and defined as:

(1) $$\frac{1}{T_{2}^{*}}=\frac{1}{T_{2}}+\gamma \Delta B_{inhom.}$$

where *γ* is the gyromagnetic ration and Δ*B_inhom_* the local magnetic field inhomogeneity. 

In practice, the *T*_2_* relaxations are observed in gradient-echo (GRE) sequences by contrast the spin-echo sequence use a 180° rephasing to avoid the effect of field inhomogeneities and record the *T*_2_ relaxation.

This technique allows the temporal or spatial (2D or 3D) reconstruction of living tissues, rich in protons, with an excellent spatial resolution, no limit in depth and without the use of ionizing radiation. The difference in image contrasts in MRI is defined by either intrinsic tissues properties (e.g., inhomogeneity in viscosity or water concentration) or operator-selected pulse sequence parameters (repetition time, TR, or echo time, TE). However, the MRI has important limitations, including a long acquisition time and the impossibility of achieving large areas in a single acquisition images (whole body for example). Another weak point of this technique is its low sensitivity, sometimes making difficult the distinction of pathological tissues. Thus, paramagnetic agents (gadolinium complexes, positive contrast) or superparamagnetic agents (iron oxide, negative contrast) are commonly used to enhance the native contrast between different tissues, which makes diagnosis more reliable. Contrast agents (CAs) dramatically shorten the *T_1_* and *T_2_* of water and their presence is easily detected in MRI images at levels as low as 0.1 mmol L^−1^. The efficiency of MRI contrast agents is determined by measuring the nuclear relaxivities *r_1,2_* defined by Equation (2):

(2) ri=[(1Ti)meas.]−(1Ti)dia.c

where (1/*T_i_*)*_meas_* is the value measured for the sample with concentration c (mmol L^−1^) of magnetic center, and (1/*T_i_*)*_dia_* refers to the nuclear relaxation rate of the diamagnetic host solution.

The first MRI *T_1_*-contrast agent, Magnevist^®^ (Bayer Schering Pharma AG, Lerverkusen, Germany), approved in 1988 is a gadolinium-based positive contrast agent stabilized with diethylenetriaminepentaacetic acid (DTPA). Even now, most of the MRI agents on the market are gadolinium-based complexes. If Magnevist^®^ remains the most used with 51% of market share, gadolinium-based complexes with different polyaminocarboxylate ligands have been developed: Omniscan^®^ (25% market share, GE Healthcare, Chicago, IL, United States) stabilized with the linear DTPA-BMA (diethylenetriamine pentaacetate bismethylamide) ligand and Dotarem^®^ (12%, Guerbet SA, Villepinte, France) with the macrocyclic DOTA (1,4,7,10-tetraazacyclododecane-1,4,7,10-tetraacetic acid) ligand [7]. However, over the past 10 years, studies have shown that gadolinium ions are released (i) by transmetallation when chelated by linear complexes (competitively with other endogenous ions such as Zn^2+^), or (ii) by acid catalyzed dissociation (even at physiological pH) when associated by macrocycles [8]. Moreover, biological ligands, such as adenosine triphosphate, block their activity [8]. Gd^3+^ ions are excreted via the renal system and therefore contraindicated for patients with renal insufficiency [9]. Therefore, since 2017, Magnevist^®^ and Omniscan^®^, in particular, have been recommended for withdrawal in Europe and the US market [10,11]. In parallel, superparamagnetic nanoparticles have been developed as a contrast agent for *T_2_*-weighted imaging. The efficiency of magnetic NPs is characterized by the largest possible value of the transverse relaxivity, *r_2_*, and *r_2_/r_1_* ratio. Iron oxide nanoparticles (magnetite, Fe_3_O_4_ and maghemite, γ-Fe_2_O_3_) are the most commonly used *T_2_*-contrast agents due to their low toxicity, remarkable magnetic properties (high magnetic susceptibility and saturation magnetization) and their great stability in biological environments [12]. As described in Table 1, negative contrast agents have been developed in the past but have since been withdrawn for commercial reasons. These contrast agents are multicore nanoassemblies of 3–5 nm magnetic nanoparticles coated with a polymer-based ligand (dextran, carboxydextran, polystyrene). One of the major shortcomings of these commercial agents was the relatively poor improvement in the contrast of imaging (*r_2_* and *r_2_/r_1_* ratio does not exceed 200 s^−1^ mM^−1^ and 20 respectively). 

To improve the effectiveness of MRI negative contrast agents, modification of their size, shape, state of assembly and surface functionalization was needed [15,16]. In particular, the use of multi-core nanoassemblies with larger spherical magnetic nanoparticles [17,18,19,20,21,22,23,24,25,26,27,28,29,30,31,32,33,34,35,36,37,38,39,40,41,42,43] has been envisaged (Table 2). Multi-core nanoparticles consist in self-assembled magnetic nanoparticles (magNAs) providing a very high effective magnetic moment per magNAs [44,45,46,47,48]. The effective magnetic moment, *μ_eff_*, is dependent on the number of nanoparticles per assembly *N*, the volume saturation magnetization *M_S_* (in A m^−1^), and the assemblies’ diameter which can, in first approximation, be assimilated to the hydrodynamic diameter *d_H_* [44,45]:

(3) $$\mu_{eff}=\sqrt{N}\frac{1}{6}\pi {d_{H}}^{3}M_{S}$$

According to the diameter *d_H_* of the contrast agent, three regimes are successively achieved (Figure 3B): first the motional average regime (MAR), then the static dephasing regime (SDR) and finally the echo-limiting regime (ELR) [19,28,49,50]. Vuong et al. [50] defined the transition from the MAR to SDR as a function of Δ*ωτ_D_* factor. The angular frequency shift experienced by a proton at the equator of the particle, Δ*ω* is calculated following Equation (4).

(4) $$\Delta \omega \;=\;\frac{{\gamma \mu }_{0}M_{S}}{3}$$

and the translational diffusion time of the proton in the magnetic field inhomogeneities, *τ_D_* is deduced from Equation (5):

(5) τD=dH24D

where the gyromagnetic factor of the proton, *γ* = 2.67513 × 10^8^ rad s^−1^ T^−1^; the magnetic permeability of vacuum, *μ_0_* = 4π × 10^7^ T m A^−1^; and the water translational diffusion time, *D* = 3.10^−9^ m^2^ s^−1^ at 37 °C.

If Δ*ωτ_D_* < 1, the *r_2_* relaxivity is described by the MAR and is expressed by Equation (6):

(6) $$r_{2}=\frac{4\gamma^{2}{d_{H}}^{2}{\left(\mu_{0}M_{S}\right)}^{2}\nu_{mat}}{405D}$$

where the iron oxide molar volume, *ν_mat_* = 1.57 × 10^−5^ and 1.50 × 10^−5^ m^3^ mol^−1^ for maghemite and magnetite respectively [50].

If Δ*ωτ_D_* > 1, the *r_2_* relaxivity followed Equation (7) of the SDR:

(7) $${r_{2}}^{*}=\;\frac{2\pi \gamma (\mu_{0}M_{S})\nu_{mat}}{9\sqrt{3}}\approx \;r_{2}$$

where r_2_* is the apparent transverse relaxivity which in addition of the *r_2_* takes into account of the local field inhomogeneity.

**Figure 4:**
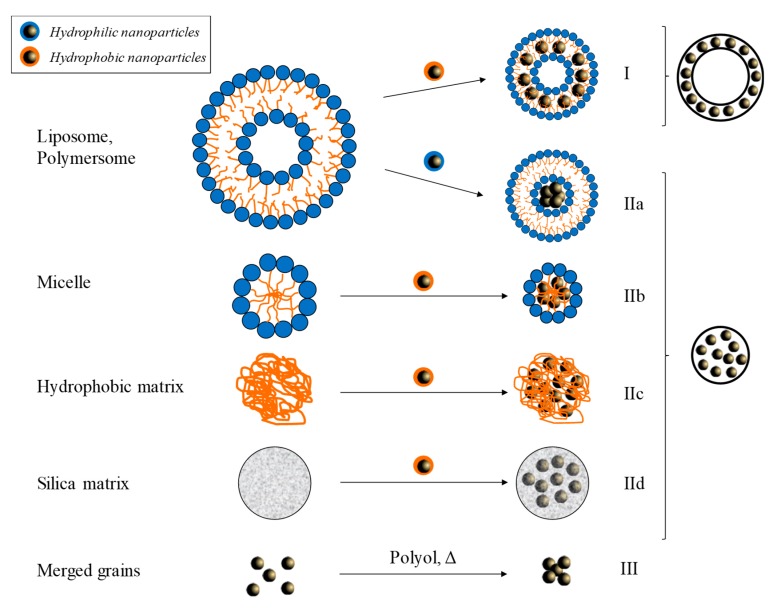
Self-assemblies of MNPs to obtain multi core magnetic nanoparticles. Three main organizations are noted: the encapsulation of MNPs in the core (type I), their dispersion on the shell of the nanoscale architecture (type II), or the merger of magnetic grains (type III).

In MAR, the relaxivity is a function of *d_H_^2^* and increases with the outer sphere diameter assimilated to *d_H_*. In SDR, the relaxivity has reached a maximum and is independent of the outer sphere diameter [28,49]. Finally, when the *d_H_* increases too much, transverse relaxivity is described by ELR. In this regime, the transverse relaxivity decreases when the size of the assemblies increases. As described in Figure 4, self-assembly of magnetic nanoparticles into silica matrix [20,27], micelle [24,25,28,30,32,34,35,38,42,43], polymersomes [17,18,30,31,36,37,39,41], or liposomes [22,26,29,40] are synthetized. Depending on the structure type, the incorporated MNPs should be hydrophilic or hydrophobic. Their dispersions can be done in two ways: in the shell (structure noted type I) or in the core (structure noted IIa-d). The MNPs used are prepared by two synthetic routes: coprecipitation (CP) or thermal decomposition (TD). The co-precipitation method is based on the hydrolysis of transition metal ions in aqueous solution [51,52]. The reaction takes place in alkaline aqueous solution with an optimal pH around 8.5–10 [53]. The nanoparticles obtained present a very broad size distribution and a size sorting is necessary [54]. The stability of these nanoparticles is insured by the surface charge: positive and in the form −OH_2_^+^ for pH between 1 and 3.5; or negative and in the form of −O^−^ for pH 9–11 [55,56]. The easy grafting of phosphoric, phosphonic or carboxylic acid derivatives hydrophilic (citrate [57], polyacrylic acid [58,59], …) or hydrophobic (oleic acid [54,56], Beycostat NB09 [18]) on the MNPs surface, allows respectively their transfer in neutral aqueous solution or organic solvent. By this method, depending on the base used and their size, the MNPs have a saturation magnetization of around 50–70 A m^2^ kg^−1^ [52,53,60] which can be increased close to the bulk material value (80 A m^2^ kg^−1^) after an hydrothermal treatment [61].

An alternative approach to the synthesis of monodisperse iron oxide nanoparticles is high-temperature organic phase decomposition of an iron precursor [62,63,64,65,66,67]. This reaction involves the thermal decomposition of a preformed organometallic precursor dissolved in a high-boiling organic solvent (usually refluxed) in the presence of capping ligands such as long-chain alkyl surfactants. The most commonly couple capping ligand/precursor used is oleic acid/iron(III) acetylacetonate. The thermal decomposition method yields very narrow particle-size distributions (standard deviation between 0.10 and 0.15) as well as excellent crystallinity and shape control. By this method, the hydrophobic MNPs possess relatively high saturation magnetization around 70–85 A m^2^ kg^−1^ [68,69,70]. Recently, several groups have shown the possibility to prepare multi-core nanoparticles without an organic matrix by a polyol route [71,72,73]. In this route the high-boiling polyol compound acts as both solvent and reductant agent of the iron precursor, an iron chloride salt. At high temperature, multicore nanoparticles with saturation magnetization in the 60–80 A m^2^ kg^−1^ range, and with different size are obtained by adjusting the used polyol (ethylene glycol, or mixture of diethylene glycol with *N*-methyldiethanolamine or sodium hydroxide) and the experimental condition [71,73,74]. Thanks to the increase of the magnetic moment per assembly, multicore MNPs provide very high value of *r_2_* up to 1400 mM^−1^ s^−1^ (Figure 4) [28]. Moreover, the clustering of MNPs significantly reduces the exchange surface between magnetic materials and water molecules inducing a decrease in the *r_1_* value. Thus, these two phenomena lead to very good *r_2_/r_1_* ratio (up to 2000) [35]. Significant values of *r_2_* (>500 mM^−1^ s^−1^) are obtained regardless of the size of the MNP used (from 6 to 16 nm), the type of organization, or the chemical route (CP, TD, polyol) adopted to prepare the magnetic nanoparticles. This observation clearly demonstrates the primacy of the total magnetic moment of assembly over the nanoparticle alone [20,26,30,31,36,38,39,41,49,74,75]. Optimal *d_H_* values are in a wide range between 70 and 250 nm depending on the systems considered. However, it is noted that this optimum is a function of the individual size of the MNP used and shifts towards low *d_H_* values when the size of the MNPs increases [28,32,35,38]. The weight percentage of iron oxide nanoparticle in the assembly (wt% IO) is also an important parameter. An increase of the value of *r_2_* and *r_2_/r_1_* ratio is observed when wt% IO increases [18,29,30]. 

### 2.2. Nanoparticles Composed of Luminophore for In Vivo Fluorescence Imaging

Fluorescence microscopy consists of visualizing emitted photons by tissues (autofluorescence) or by exogenous luminophores—after an excitation of higher energy. The use of exogenous luminophores allows the acquisition of specific physiological tissue and cell imagery, quickly and without ionizing radiation [76,77,78]. In this way, cells activity is investigated at different levels: investigation of Ca^2+^-pathway [79], or tracking cellular metabolites [80], biological macromolecules [81] as proteins [82]. The sensitivity of the technique is related to the specificity of the luminophore: its quantum yield and its brightness [83]. The fluorescence quantum yield, *Φ*, is defined as the number of emitted photons per the number of absorbed photons. Its calculation requires a reference as described below [84]:

(8) $$\phi_{x}=\phi_{ref}\cdot \;\frac{I_{x}}{I_{ref}}\cdot \frac{A_{ref}}{A_{x}}\cdot \;\frac{n_{x}^{2}}{n_{ref}^{2}}$$

where *Φ_ref_,* the known fluorescence quantum yield of a reference luminophore; *I_x_ and I_ref_* are the integral of the corrected emission signal at the same excitation wavelength of the luminophore and the reference, respectively. *A_x_* and *A_ref_* are the absorbance values at the excitation wavelength of the luminophore and the reference, respectively. *n_x_* and *n_ref_* the solvant refractive index of the luminophore and the reference respectively. 

The brightness, *B*, is defined as the product of the molar absorption coefficient at the excitation wavelength, *ε(λ_ex_.)*, and the fluorescent quantum yield, *Φ*:

(9) $$B=\;\phi_{x\;}\cdot \;\varepsilon \left(\lambda ex.\right)$$

**Figure 5:**
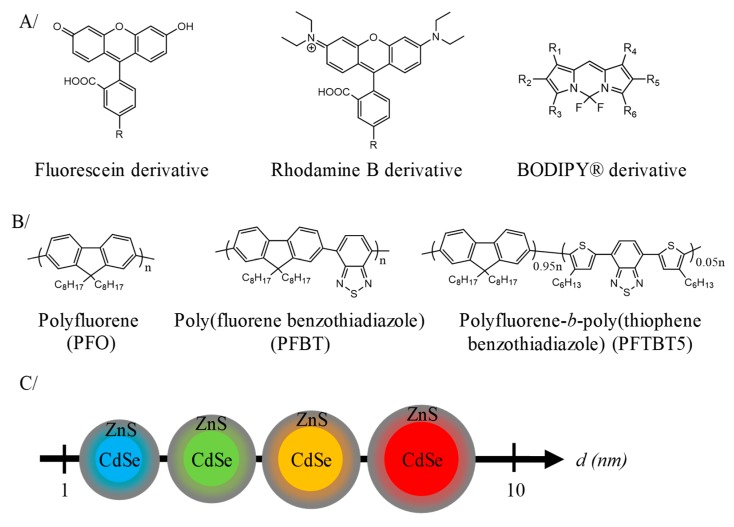
Description of some luminophores. (**A**) Chemical formula of the main families of fluorophore. (**B**) Structure of Pdots based on polyfluorene. (**C**) Cartoon illustrating the effect of QDots size on their luminescent properties.

This value is the analytical parameter defining the sensitivity of the luminophore. A large panel of luminescent compounds are developed including, fluorescent molecules, fluorescent proteins, polymer dots (Pdots) or quantum dots (Qdots) [83,85,86,87]. Small molecules such as BODIPY, rhodamine or fluorescein derivatives (Figure 5A), are easily accessible, biocompatible and present tunable emissive properties from 200 to 800 nm by chemical derivation [88,89]. Moreover, these derivatives can be modified to probe organelles or cellular membranes [90]. Thus, mitochondria or lysosomal compartments are stained respectively by Rhodamine 123 (*λ_max_^em^* = 529 nm) [91] or LysoTracker^®^ Red (*λ_max_^em^* = 590 nm) [92]. Nucleus staining is achieved by fluorophore binding to the base pairs of double-stranded DNA without pair specificity for propidium iodide dye or with a specificity of A-T regions for 4′,6-diamidino-2-phenylindole (DAPI) and Hoechst 33342 dye [93]. The blue (*λ_max_^em^* = 450–490 nm) labeling of the nuclear DNA can be conducted by DAPI for fixed cells or Hoechst 33342 for live cells [93]. Propidium iodide (*λ_max_^em^* = 617 nm) stains preferentially the nucleus of permeable cells and is used as necrotic label. These systems generally have a relatively high brightness around 10^4^–10^5^ L mol^−1^ cm^−1^. Nanoprecipitation of hydrophobic fluorophore gives fluorescent organic nanoparticles (FON) [94,95,96,97,98,99]. These systems are composed of a multifold of dyes (10^4^ to 10^5^) that are neither covalently linked nor diluted in an inert matrix, which yields highly bright structures (10^6^–10^7^ M^−1^ cm^−1^) [99,100]. Under mono- or biphotonic excitation, in cellulo imaging showed that these nanoarchitectures appeared as bright as quantum dots, allowing their use for tracing cancer cells and macrophages [101]. Fluorescent proteins have similar brightness of non-assembled fluorophore and allow better biocompatibility [102]. Among these proteins, the green fluorescent protein (GFP) is the more studied [103,104,105,106,107]. This green protein (*λ_max_^em^* = 510 nm) has been isolated in 1960 from the jellyfish, *Aequorea victoria*. The discovery and development of GFP led O. Shimomura, M. Chalfie and R. Y. Tsien to the 2008 Nobel Prize. However, it has low photostability and a high cost of production [108]. Pdots are appreciated for their brightness properties (10^5^–10^6^ L mol^−1^ cm^−1^) and their low cytotoxicity [109]. They are conjugated polymers based on aromatic rings (Figure 5B) as poly[2,5-di(3,7-dimethyloctyl) phenylene-1,4-ethynylene] (PPE), poly[2-methoxy-5-(2-ethylhexyloxy)-1,4-phenylenevinylene] (MEH-PPV), polyfluorene derivitative as poly(9,9-dioctylfluorenyl-2,7-diyl), PFO (*λ_max_^em^* = 435 nm); poly[(9,9-dioctyl-2,7-divinylene-fluorenylene)-alt-co-(2-methoxy-5-(2-ethylhexyloxy)-1,4-phenylene)], PFPV (*λ_max_^em^* = 510 nm); poly[(9,9-dioctylfluorenyl-2,7-diyl)-co-(1,4-benzo-{2,1′,3}-thiadiazole)], PFBT (*λ_max_^em^* = 545 nm) [110,111,112,113,114,115,116,117]. By controlling the operating parameters, self-assembly of these polymers gives nanoparticles, (typically from 10 to 50 nm) [99,110,111,112,113,114,115,116,117]. Nevertheless, a decrease in quantum yield of nearly 70% is noted when the size of a self-assembly of polybutylene terephthalate (PBT) goes from 10 to 40 nm [110,111,112,113,114,115,116,117]. Finally, Qdots are semi-conducting nanocrystals with high brightness (10^6^ L mol^−1^ cm^−1^), high photostability and narrow emission signal [118,119,120]. As described in Figure 5C, the emission wavelength depends on the size of the crystal, and can vary from blue (380 to 440 nm) for smaller Qdots (~2 nm diameter) to red (605 to 630 nm) for larger particles (~5 nm diameter) [121]. Actually, the core shell CdSe/ZnS Qdots are the most common with a 90% luminescence quantum yield [122,123]. Yet, toxicity problems due to the presence of heavy metals and photoblinking are generally noted although efforts have recently been made to avoid these pitfalls [124,125,126]. 

Moreover, its application for in vivo studies has been limited for a long time because of (i) tissue autofluorescence, which decreases the signal-to-noise ratio (and therefore sensitivity), (ii) the absorption of photons by the biological tissues, which respectively induce an important depth limit. To overcome this problem, fluorophores emitting in the near infra-red have been developed. Indeed, in this area, the overall absorption of biological tissues is minimal: it is called the first biological window (700–950 nm) [127]. Thus, near infra-red fluorescence imaging (NIRF) faces limited light penetration into biological tissues, is used in preclinical small rodents studies, and has revealed a very promising potential for guided surgery [128,129]. If the association of two active units within the same system is very promising for multimodal imaging, precautions have to be taken to preserve the properties of each unit. This is especially true for fluorescent systems where magnetic nanoparticles units can deactivate or absorb part of the emitted light. The way to assemble these units is therefore a crucial parameter.

## 3. Magneto-Fluorescent Nanosystems

### 3.1. Association by Covalent Bonding (Nanoparticles)

**Figure 6:**
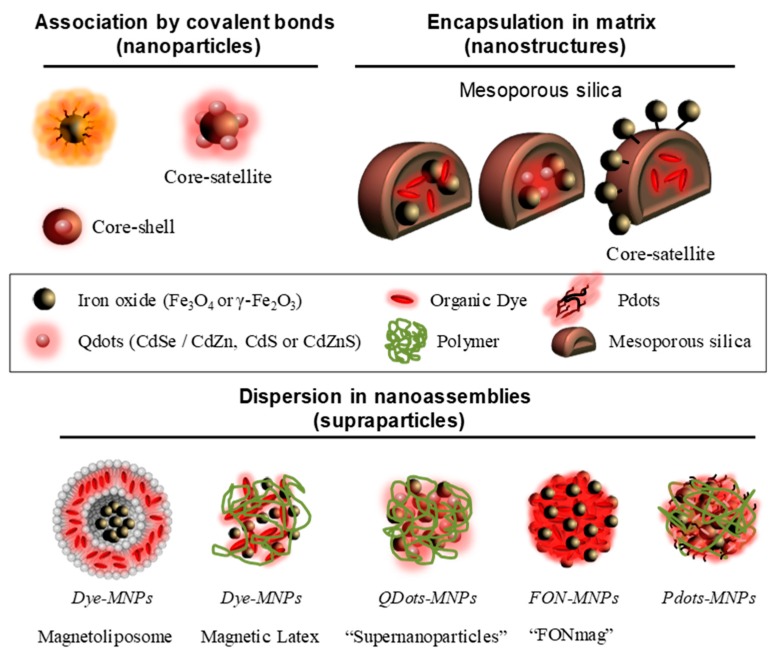
Schematic representation of magneto-fluorescent nanosystems.

Among the different association methods, grafting fluorescent entities on the surface of magnetic nanoparticles represents a simple approach (Figure 6) [130,131,132,133]. For example, fluorescent dyes (rhodamine B, *λ_max_^em^* = 578 nm or fluorescein derivatives, *λ_max_^em^* = 516 nm) are coupled to iron oxide surface [130]. These nanoparticles allow in cellulo motions of endosomes to be followed when exposed to a magnetic field gradient. Near-infrared (NIR) dyes have been also grafted as IR-820 cyanine derivative (*λ_max_^em^* = 900 nm) [132] or dialkyl carbocyanine (*λ_max_^em^* = 780 nm) [133] to obtain fluorescent systems emitting in the first biological window. Iron oxide multicore nanoparticles assembled by hydrophilic polymer have been envisaged to improve the MRI contrast agent properties [131,133]. Thus, the use of multicore Ferahme^®^ iron oxide nanoparticles (*d_core_* = 6–7 nm, *d_H_* = 17–31 nm) coupled to TO-PRO^®^-1 (*λ_max_^em^* = 531 nm) gives bifunctional nanoparticles with a transverse relaxivity, *r_2_* = 122 mM^−1^ s^−1^ (0.47 T, *r_2_/r_1_* = 5) [131]. This transverse relaxivity is still improved using 8 nm iron oxide nanoparticles (*r_2_* = 202 mM^−1^ s^−1^, *r_2_/r_1_* = 3.8 at 0.47 T) embedded in polyacrylic acid matrix (*d_H_* = 90 nm) [133]. Moreover, these systems present NIR-emissive dye using a dialkylcarbocyanine as fluorophores (*λ_max_^em^* in the region 751/780 nm and *ε* > 125,000 cm^−1^ M^−1^). The association of QDots, known to be brighter than small molecules, with magnetic nanoparticles is also envisaged [134,135,136,137,138,139,140]. The most common approach used is to prepare core–satellite systems. In this approach the core is composed of iron oxide nanoparticles surrounded by quantum dots (usually CdSe/ZnS) [134,135,136,140]. Pahari et al. have recently described an invert strategy where quantum dots (3.2 nm CdSe nanoparticles) are in the core and a shell of iron oxide is growned around (thickness of 1.3 nm) [139]. By this approach, a very good transverse relaxivity (*r_2_* = 304 mM^−1^ s^−1^ at 9.4 T) is noted. Although these systems are easily synthetized, the close proximity between the luminophores and the metal core leads to a strong emission quenching. Indeed, electronic energy or electron transfers between both entities can take place while iron oxide nanoparticles significantly absorb at wavelengths less than 450 nm. The choice of fluorophores and their distance from the metallic core will therefore be essential. Moreover, direct exposure of fluorophores to the surrounding environment can modify the emissive properties of the system. Finally, the requirement of high colloidal stability of the final nanoassembly in aqueous solution excludes extensive grafting of fluorescent entities, especially if the latter are organic and hydrophobic. All together, these limitations produce low-emissive imaging agents.

### 3.2. Encapsulation in Silica Matrix (Nanostructure)

To protect the luminophores from quenching by the surrounding medium, the encapsulation of magnetic nanoparticles (γ-Fe_2_O_3_) and fluorescent units (small molecules, e.g., rhodamine or FITC derivatives; [141,142,143,144,145] or Qdots like CdSe/CdZn, CdS or CdZnS [146,147,148,149,150]) in mesoporous silica matrices has been envisaged (Figure 6). The encapsulation of magnetic nanoparticles and quantum dots leads to interactions between these two active units. This interaction induces (i) an increase of the magnetic anisotropy, (ii) a blue-shift of the fluorescence emission and (iii) a decrease of the quantum yield [146,149]. Silica-doped with organic dye core surrounded by magnetic nanoparticles (core-satellite assemblies) are also envisaged [141,142]. In these structures, the combination of several magnetic nanoparticles has the effect of drastically increasing the *r_2_* value in comparison of magnetic nanoparticles alone. Lee et al. describe an *r_2_* increase from 26.8 to 76.2 mM^−1^ s^−1^ (1.5 T) [141], and another study shows a rise from 116 to 397 mM^−1^ s^−1^ (9.4 T) [142]. These silica matrices show low cytotoxicity but provide only small amounts of encapsulated active units. In addition, although the fluorescent entities are protected from the external environment by encapsulation, they can diffuse freely outside the porous matrix as they are not covalently attached. To counter this phenomenon, hydrophobic fluorescent units amenable to self-assemble have been proposed to impart the magneto-fluorescent nanosystems with better structural stability and reduced dye leakage.

### 3.3. Dispersion in Nanoassemblies (Supraparticles)

The use of magneto-fluorescent nanoassemblies provides generally biodegradable systems that advantageously avoid bioaccumulation. In this context, several molecular matrices are envisaged composed of polymers [151,152,153,154,155,156,157,158,159], lipids [160,161,162,163], PDots [164,165,166,167] or organic molecule [168,169,170,171,172] (Figure 6). In this type of organization we will distinguish the assemblies with inert matrices of those composed of active units. Inert matrices are mainly composed of lipids or polymers. For instance, magnetic nanoparticles can be encapsulated in liposomes, and subsequently functionalized by a fluorescent molecule, here rhodamine [160]. The magnetofluorescent liposome exhibits good *T_2_*-contrast agent properties with *r_2_* = 268 mM^−1^ s^−1^ at 4.7 T (*r_2_/r_1_* = 85). Another approach is based on the use of polymers to combine magnetic nanoparticles and fluorescent entities. In the work of R. K. Prud’homme et al., polyethylene glycol has also been used to assemble hydrophobic NIR fluorophores (*λ_max_^em^* = 800 nm), tris-(porphyrinate) zinc (II), and magnetic nanoparticles [153]. The authors show an increase in *r_2_* from 66 to 533 mM^−1^ s^−1^ as the wt% IO increases from 4 to 16% [153]. Bawendi et al. describe the association of quantum dots and densely packed magnetic nanoparticles into “supernanoparticles” thanks to poly(vinylpyrrolidone) (PVP) ethylene glycol (EG) [152]. These assemblies (*d_H_* = 120 nm) display a high *r_2_* value of 402.7 mM^−1^ s^−1^ at 9.4 T [152]. In the three last cases, the effectiveness of these multimodal structures has been demonstrated in murine models. Correlative treatments of the MRI and fluorescence signals have proved the preservation of the in vivo integrity of the nanoassemblies and validate the design of multimodal nanostructures.

The second type of self-assembled systems implies functional units as molecular bricks (Figure 6), thus limiting the number of organic species administrated in vivo. Hyeon et al. have assembled magnetic nanoparticles with a polyethylene glycol block polymer [157]. This polymer is functionalized with an imidazole derivative and fluorescent porphyrins (chlorin e6) whose emission is deactivated upon dye self-assembling [157]. The imidazole derivative is a pH-sensitive group which allows the disintegration of the nanostructure in the tumor medium (acidic pH) and leading to the reappearance of fluorescence. In this system (*d_H_* = 70 nm), the self-assembly of 3 nm iron oxide nanoparticles provides a transverse relaxivity *r_2_* = 44 mM^−1^ s^−1^ at 1.5 T (*r_2_/r_1_* = 13.3). Nanoassemblies incorporating Pdots and magnetic nanoparticles into phospholipid micelles improve relaxivity properties (*r_2_* = 152mM^−1^ s^−1^ at 3.0 T) and enhance MRI contrast efficiency thereof [167]. Moreover, for these systems, important brightness and photostability under irradiation of the fluorophores have been demonstrated during in cellulo fluorescence microscopy [167]. The use of small hydrophobic molecules with iron oxide nanoparticles chelating functions is another effective approach to obtain magnetofluorescent systems [168,169,170,171,172]. In these systems, the fluorescent core composed of 10^5^ dyes is surrounded by magnetic nanoparticles shell [168]. This architecture deals with a very effective dual-mode contrast agent with a brightness around 10^7^ L mol^−1^ cm^−1^ and transverse relaxivity *r_2_* = 238 mM^−1^ s^−1^ (0.47 T) [168]. This contrast agent displays excellent properties in liver imaging on small rodents both as a cellular label and as in vivo follow-up [168]. In cellulo stability of these systems could be controlled by varying stabilizing ligands [170]. The use of polyacrylic acid allows a very cohesive architecture, when the stabilization by citrate ions allows a dissociation [170]. These systems have been functionalized with polyethylene glycol-based copolymers to increase their circulation time [172]. Moreover, it has been shown that the presence of a hydrophobic tail in the copolymer increases the *r_2_/r_1_* two times compared to those which are without one [172]. 

## 4. Conclusions and Future Outlook

The combination of magnetic and fluorescent units into a single nanomaterial provides imaging agents from in cellulo (fluorescence imaging) to in vivo (MRI) experiments through the imaging of small rodents (colocalization of fluorescence and MR signal). These very promising nano-objects must be carefully synthesized to preserve the physical properties of each active unit. We have assessed three approaches to address the issue: (i) association by covalent bonding, (ii) encapsulation of matrix, (iii) dispersion in nanoassemblies. The three systems allow multicore magnetic nanoparticles to be obtained. This configuration allows to obtain high transverse relaxivity value (*r_2_* > 200 mM^−1^.s^−1^). The first and simplest method provides nevertheless magneto-fluorescent systems with low brightness. The second significantly improves the brightness but produces systems that are not stable over time. Finally, the third method seems to be, at the moment, the most promising because it provides ultra-bright, high MRI sensitive and stable nanoassemblies allowing a long-term follow-up. Thanks to these structures, the encapsulation of drugs can also be envisaged. Indeed, drug delivery by supramolecular nanoassemblies (supraparticles) allows to associate a wide diversity of active units simply assembled by weak bonding. These systems offer combinatorial modularity, tunable properties and biodegradability. Thus, supramolecular nanoassemblies appear as very promising drug carriers able to vectorize a large amount of drug, monitor its delivery thanks to imaging agents, and control its release with remote tunable stimuli.

In order to transfer these promising multimodal nano-objects from the laboratory to the clinic, a number of bottlenecks still need to be addressed. The in vivo biodistribution and fate of nano-systems remain the main obstacles. These points are crucial for any object designed for nanomedicine but are still more complicated in the case of a heterogeneous object and in which each unit is capable of behaving differently in a biological environment. Thus, for these complex structures it is necessary to ensure their in vivo integrity during the imaging procedures. In addition, it is necessary to know the long-term fate of all the active units i.e., their possible bioaccumulation or long-term toxicity. Thus, a back and forth between chemist and biologist to adapt and follow in vivo these multimodal objects is required. However, the multidisciplinary approaches implemented in numerous research programs will be able to overcome these limitations.

## Figures and Tables

**Table 1:** T_2_ contrast agents that have been proposed to the European and American markets. *d_H_* is the hydrodynamic diameter of the nanoparticle assembly. *r_2_* is given at 1.5 T and 37 °C [13,14].

Name	Classe	*d_H_* in nm/Coating	*r_2_* in s^−1^ mM^−1^/(*r_2_/r_1_*)	Approval (withdrawn)	Company
Endorem^®^ or Feridex I.V	ferumoxides	120–180/dextran 10 kDa	158 (16)	1994 (2012) or 1996 (2008)	Guerbet S.A. or Berlex Laboratories
Sinerem^®^ or Combidex ^®^	ferumoxtran-10	20–40/dextran 10 kDa	88 (5)	n.a. (2007) or 2005 (2007)	Guerbet S.A. or AMAG pharmaceuticals, Inc.
Resovist^®^	ferucarbotran	45–60/carboxydextran 1.8 kDa	189 (19)	2001 (2009)	Bayer Healthcare
Feraheme^®^	ferumoxytol	30/semi-synthetic carbohydrate	89 (6)	2009	AMAG pharmaceuticals, Inc.
Lumirem^®^ or GastroMARK^®^	ferumoxsil	400/poly [N-(2-aminoethyl)-3-aminopropyl]siloxane	47 (23)	1993 (2014) or 1996 (2010)	Guerbet S.A. or AMAG pharmaceuticals, Inc.

**Table 2:** Properties of self-assembled magnetic nanoparticles designed as MRI contrast agent.

Type	Dispersant	*d_core_* in nm	Synthesis Route (Provider)	*d_H_* in nm	wt% IO	Field/T	*r_2_* in mM^−1^ s^−1^ (r_2_/r_1_)	Ref.
I	PAA-*b*-PS	5.6	TD	513	25	1.41	295(n.a)	[39]
		6.4		400			378 (n.a)	
		10.8		300			561 (n.a)	
		15.5		241			555 (n.a)	
I	PTMC-*b*-PGA	6.3	CP	50	20	4.7	81 (29)	[18]
				45	35		134 (37)	
				47	50		173 (48)	
				52	70		182 (52)	
I	PTMC-*b*-PGA	6–7	CP	125	5	1.41	71 (14)	[30]
		6–7		109	51.6		114 (25)	
		8–10		67	33.8		128 (22)	
		8–10		79	50.5		167 (25)	
		10–15		87	5.1		219 (71)	
		10–15		148	20		280 (103)	
I	PR-PAA in organosilica matrice	6	TD	76	10	3	642 (n.a)	[31]
I	PEG-*b*-PCL-*b*-PAA	1.9	CP	140	3	1.41	108 (n.a)	[17]
I	Pluronic^®^ L-121	10	n.a. (Webcraft GmbH)	126	7.1	1.41	682 (68)	[41]
I	DOPG or DOPC	13.8	CP	90	n.a	1	166 (~20)	[26]
				110			919 (~20)	
I	folic acid-PGA-*b*-PCL	7	CP	174	n.a	1.41	612 (20)	[36]
I	PEG-*b*-poly(tert-butyl acrylate-stat-PAA	6	CP	175	4.8	3	211 (n.a)	[37]
IIa	EPC and DSPE-PEG-methoxy 2000	7.7	CP	16	100	0.47	108 (3)	[29]
				200	35^1^		116 (6)	
				195	63^1^		130 (17)	
IIb	SDS	9.1	TD (Ferrotec)	53	75	3	295 (n.a)	[32]
				80			350 (n.a)	
				99			410 (n.a)	
IIb	PCL-*b*-PEG	4	TD	17	12.4	1.5	25 (19)	[43]
		4		75	19.5		169 (58)	
		8		97	38.1		318 (199)	
		16		110	54.2		471 (236)	
IIb	PEG-*b*-PAA	8.2	TD	105	34	1.41	255 (6)	[38]
				139			444 (6)	
				181			604 (14)	
IIb	PI-*b*-PEG	8	TD	54	n.a.	1.41	131 (n.a)	[35]
				89			250 (n.a)	
				96			353 (n.a)	
				216			16 (n.a)	
IIb	PEG-*b*-PBLG	6–7	CP	157	5	1.41	180 (90)	[30]
		8–10		63	20.1		95 (20)	
		8–10		73	25		90 (19)	
		8–10		87	29.7		105 30)	
		10–15		109	20		500 (126)	
IIb	GCPQ	4.8	TD	140	n.a	1	52 (79)	[24]
IIb	PEG-*b*-PLGA	7	TD	73	41	1.5	333 (n.a)	[25]
IIb	PTEA-*b*-PAM	6.3	CP	11	1^2^	0.47	39 (2)	[34]
				70	32^2^		74 (3)	
				170	150^2^		162 (9)	
IIb	PEI-*b*-PCL-*b*-PEG	4	TD	60	n.a	1.41	20 (n.a)	[28]
		4		130			56 (n.a)	
		4		170			72 (n.a)	
		7.5		45			100 (n.a)	
		7.5		80			200 (n.a)	
		7.5		130			175 (n.a)	
		8.7		45			115 (n.a)	
		8.7		80			235 (n.a)	
		8.7		180			70 (n.a)	
		9.8		55			50 (n.a)	
		9.8		120			375 (n.a)	
		9.8		190			350 (n.a)	
		11.8		50			200 (n.a)	
		11.8		100			420 (n.a)	
		11.8		220			100 (n.a)	
IIc	lauric acid-irinotecan prodrug	20	TD (Sigma-Aldrich)	117	6	7	189 (n.a)	[33]
IId	silica	7	CP	24	25	7	179 (n.a)	[20]
				41	27		779 (n.a)	
				26	42		1395 (n.a)	
IId	silica	6.1	TD	160	5	0.47	148 (510)	[27]
				120	7.4		164 (607)	
				313	5.9		326 (1917)	
III	PAA	n.a	Polyol	79	100	1.41	405 (n.a)	[75]
				122			508 (n.a)	
III	PAA	7.5	Polyol	15	100	0.47	247 (n.a)	[49]
		9		30			340 (n.a)	
		11.6		50			364 (n.a)	
		19.7		100			100 (n.a)	
III	PAA	15.6	Polyol	37	100	0.23	361 (3.5)	[74]
		12		38.5			365 (3.4)	
		13.5		44.3			319 (3.1)	
		11		27			289 (3.1)	

^1^ molar ratio; ^2^ Number of nanoparticles per assembly. Abbreviation: IO: iron oxide, NF: nanoflowers, TD: thermal decomposition, CP: co-precipitation, PAA: poly(acrylic acid), PS: polystyrene, PTMC: poly(trimethylene carbonate), PGA: poly(*L*-glutamic acid), PEG: poly(ethylene oxide) or polyethylene glycol, PCL: poly(ε-caprolactone), PLGA: poly(lactic-co-glycolic acid), PEI: poly(ethylene imine), PI: polyisoprene, PTEA: poly(trimethylammonium ethylacrylate methyl sulfate), PAM: poly(acrylamide), PBLG: poly(γ-benzyl-*L*-glutamate), PR: polyrotaxane, GCPQ: *N*-palmitoyl-*N*-monomethyl-*N*-*N*-dimethyl-*N*-*N*-*N*-trimethyl-6-O-glycolchitosan, EPC: egg *L*-α-phosphatidylcholine, DSPE-PEG-methoxy 2000: 1,2-distearoyl-sn-glycero-3-phosphoethanolamine-N-[methoxy(polyethylene glycol)-2000] (ammonium salt), DOPG: 1,2-dioleoyl-sn-glycero-3-[phospho-rac-(1-glycerol)] (sodium salt), DOPC: 1,2-dioleoyl-sn-glycero-3-phosphocholine, SDS: Sodium dodecyl sulfate.
